# Contrast-enhanced ultrasonography for assessing histopathology in pediatric immunoglobulin A nephropathy and Henoch–Schönlein purpura nephritis

**DOI:** 10.1007/s00247-022-05399-3

**Published:** 2022-06-13

**Authors:** Hejia Zhang, Qinglin Liu, Zhi Chen, Xingfeng Yao, Chen Ling, Lei Lei, Xiaoman Wang, Xiaorong Liu, Xiangmei Chen

**Affiliations:** 1 Department of Nephrology, Beijing children’s Hospital, Capital Medical University, Beijing, China; 2 National Center for Children’s Health, Beijing, China; 3 Department of Ultrasound, Beijing Children’s Hospital, Capital Medical University, Beijing, China; 4 Department of Pathology, Beijing Children’s Hospital, Capital Medical University, Beijing, China; 5 Department of Nephrology, Beijing Children’s Hospital, Beijing West District Nan Li Shi Lu 56th, Beijing, 100045 China; 6 Department of Nephrology, Chinese PLA General Hospital, Chinese PLA Institute of Nephrology, State Key Laboratory of Kidney Diseases, National Clinical Research Center for Kidney Diseases, Beijing, China

**Keywords:** Children, Contrast-enhanced ultrasound, Henoch–Schönlein purpura nephritis, Immunoglobulin A nephropathy, Kidney, Pathology, Ultrasound

## Abstract

**Background:**

Glomerular disease, including immunoglobulin A nephropathy (IgAN) and Henoch–Schönlein purpura nephritis, is one of the most common kidney diseases in children. The diagnosis of these diseases depends on pathological biopsy, although this procedure is seriously limited by its invasive and high-risk nature.

**Objective:**

To investigate the potential of contrast-enhanced ultrasonography (CEUS) for evaluating the histopathological severity of IgAN and Henoch–Schönlein purpura nephritis (HSPN).

**Materials and methods:**

We investigated a total of 13 children with IgAN and 12 children with HSPN confirmed by renal histopathology. We reevaluated the pathological lesions of the children according to the Oxford classification and the Lee grading system and then all the children underwent CEUS. Using SonoLiver software, we constructed time–intensity curves of CEUS for regions of interest in the renal cortex. We analyzed CEUS quantitative parameters for IgAN and HSPN and used Spearman correlation analysis to examine the correlation between CEUS parameters and clinicopathological indexes in the study cohort.

**Results:**

The CEUS parameters rise time (RT) and time to peak (TTP) were significantly higher in children with Lee grade IV than in those with Lee grades II or III. Spearman correlation analysis revealed a positive correlation between rise time and time to peak with Lee grade in the overall cohort of children, and a positive correlation between rise time and time to peak and severity of crescents in the Oxford classification scoring system.

**Conclusion:**

Contrast-enhanced US may be used as a noninvasive imaging technique to evaluate the severity of renal pathology and formation of crescents in children with IgAN and HSPN.

**Supplementary Information:**

The online version contains supplementary material available at 10.1007/s00247-022-05399-3.

## Introduction

Glomerular disease is one of the most common kidney diseases in children, and immunoglobulin A nephropathy (IgAN) and Henoch–Schönlein purpura nephritis (HSPN, also called IgA vasculitis) are the most common primary and secondary glomerular diseases, respectively. These two diseases have similar clinical manifestations and share the same histological feature of predominant glomerular deposits of IgA, mostly represented by the subclass IgA1 [1]. The diagnosis of these diseases depends on the findings of a pathological biopsy, although this procedure is seriously limited by its invasive and high-risk nature. Consequently, assessing the degree of renal damage using an easy and noninvasive method to guide clinical therapy is both essential and important.

The contrast-enhanced ultrasound (CEUS) technique, which uses a microbubble-based contrast agent, has been used widely for quantitative evaluation of the microvascular perfusion of parenchymal organs. CEUS is a noninvasive, safe, well-tolerated and reproducible procedure, and especially because it does not cause nephrotoxicity, it is currently used to assess renal microvascular perfusion in people with chronic kidney injury, renal mass characterization, renal intervention and renal transplantation [2–5].

However, published literature specifically assessing the use of CEUS for evaluating renal pathology is limited, especially in children. The purpose of this study was to investigate the value of CEUS quantitative parameters for predicting the severity of pathological damage in children with IgAN and HSPN, and to evaluate whether CEUS has potential as a new imaging technique to evaluate glomerular diseases.

## Materials and methods

### Study design and patients involved

The ethics committee of our hospital (No. 2018–183) approved this study and we obtained parental consent. All children who met the diagnostic criteria of IgAN and HSPN confirmed by renal biopsies carried out at the Department of Nephrology between August 2019 and August 2021 were enrolled in the study [1, 6]. Children who did not accept the risk of CEUS were excluded from the study, as well as those with poor breathing cooperation, pulmonary embolism, cardiac failure or severe allergic conditions. Finally, 13 children with IgAN and 12 children with HSPN were included. All children underwent CEUS immediately after their diagnosis and had routine examinations that included a urine test, quantification of 24-h urine protein excretion and serum creatinine, urea nitrogen and albumin levels.

### Contrast-enhanced ultrasound examination

All children underwent conventional US and CEUS examination using a Philips iU22 color Doppler US diagnostic instrument (Philips Healthcare, Bothell, WA) with a 1–5-MHz convex transducer. All the scanning procedures were performed by one radiologist (Q.L.) with more than 10 years of diagnostic experience. First, the child was placed in the lateral decubitus position and then a conventional US was performed to observe the size, structure and blood flow distribution of the kidneys and to measure the maximum long-axis view of the kidney. CEUS was then performed on both kidneys. The contrast agent was SonoVue (Bracco Suisse SA, Geneva, Switzerland), which contained sulfur hexafluoride microbubbles stabilized by a phospholipid shell [7]. After identifying the optimal long-axis view of the kidney, the radiologist manually kept the transducer in a stable position and instructed the child to breathe shallowly to minimize the variation caused by motion. SonoVue was mixed with 5-mL saline before use. Then 0.03 mL/kg (maximum dose 2.4 mL) of the suspension was administered via an antecubital vein as a bolus injection, followed immediately by a flush of 5 mL of saline solution. Continuous imaging was captured and observed in real time for 3 min after the injection of SonoVue. All images and video clips were stored in Digital Imaging and Communications in Medicine (DICOM) format for subsequent analysis.

### Image analysis

The CEUS images were analyzed using off-line SonoLiver quantitative analysis software (TomTec Imaging Systems, Munich, Germany; and Bracco, Geneva, Switzerland) by a radiologist (L.L) with more than 6 years of experience in image analysis. The regions of interest (ROIs) were located in the renal cortical area with subsequent automatic generation of a time–intensity curve. All ROIs consisted of > 100 pixels and the regions were the same as biopsy. The function of motion compensation was used, which enabled the SonoLiver software to automatically minimize the influence of breathing. ﻿﻿Perfusion parameters including of maximum intensity (IMAX, the percentage ratio of intensity in the lesion to that in the reference at the highest point of the perfusion process), rise time (RT, time between 10% and 90% of IMAX), time to peak (TTP), mean transit time corresponding to the center of gravity (mTT) and the quality of fit (QOF) were obtained from the time–intensity curve curves. A QOF means the quality of fit between raw data and theoretical curve, with greater than 75% considered as credible for the results. For every ROI, we repeated the analysis three times and calculated the mean value of the perfusion parameters.

### Renal biopsy and histopathology

The renal biopsies were performed under US guidance, with the puncture point being the inferior pole of the right kidney. All children or their guardians signed informed consent before the operation. All biopsy samples were sent for pathological examination that included immunofluorescence, light microscopy and electron microscopy. Sections for light microscopy were stained by Masson trichrome, hematoxylin–eosin, periodic acid–Schiff (PAS) and periodic acid–silver methenamine (PASM). A renal pathologist (X.Y., with more than 15 years of experience in renal pathological diagnosis) was responsible for histopathological diagnosis.

Because of the similarities between HSPN and IgAN etiology, the clinical manifestations and pathological findings in the renal pathology of children in the HSPN group was also re-evaluated according to the Lee grading standard and the updated Oxford classification [8, 9]. In the Lee grading standard [10] grade I, the glomeruli are mostly normal, with occasional mild mesangial thickening (segmental) with or without hypercellularity. In grade II, less than half of the glomeruli show localized mesangial proliferation and sclerosis, with rare or small crescents. Grade III is diffuse mesangial proliferation and thickening with focal and segmental variation, occasional small crescents and adhesions, focal interstitial edema and infiltrates occasionally present. Grade IV is marked diffuse mesangial proliferation and sclerosis, with crescents present in up to 45% of glomeruli, frequent partial or total glomerulosclerosis, tubular atrophy, interstitial inflammation and occasional interstitial foam cells. Grade V is similar to IV but more severe, with crescents present in more than 45% of glomeruli, and tubular and interstitial changes similar to IV but more severe.

The Oxford classification (MEST-C score) [6] measures the mesangial score (M0 ≤ 50%, M1 > 50%), endocapillary hypercellularity (E0 absent, E1 present), segmental glomerulosclerosis (S0 absent, S1 present), tubular atrophy/interstitial fibrosis (T0 ≤ 25%, T1 26–50%, T2 > 50%), and cellular/fibrocellular crescents formation (C0 absent, C1 < 25%, C2 ≥ 25%).

### Statistical analysis

Statistical analysis was performed using SPSS statistical software version 24.0 (﻿IBM, Armonk, NY). Data were expressed as mean value ± standard deviations. We used a *t*-test to analyze for comparisons between two groups. We used one-way analysis of variance (ANOVA) followed by least significant difference (LSD) test to analyze the comparisons between different groups. The counted data were expressed as the number of cases and the differences among groups were analyzed by using chi-square test. Correlation analysis was performed using the Spearman rank correlation analysis. A two-sided *P-*value of less than 0.05 was considered a statistically significant difference.

## Results

### Demographics and data analysis

Initially 27 children were identified; however, 2 were excluded due to poor respiratory coordination, and therefore 25 children were recruited to the study in total. The demographics of the two patient groups are summarized in Table 1. The IgAN group consisted of 13 children (8 boys and 5 girls) with a mean age (± standard deviation) of 11.3 ± 2.6 years, while the HSPN group consisted of 12 children (8 boys and 4 girls) with a mean age of 10.4 ± 3.9 years. Serum urea nitrogen level was significantly higher in children in the IgAN group than in the HSPN group (*P* = 0.001) and systolic blood pressure in the IgAN group was significantly higher than in the HSPN group (*P* = 0.03). There were no statistically significant differences in the other clinical characteristics including gender, age, medical history, serum creatinine and albumin levels, urinary protein excretion, or the level of hematuria. The Lee grading standard and Oxford classification showed that the distribution of pathological variables was not statistically different between groups (Online Supplementary Materials 1, 2, 3, 4, 5 and 6).

**Table 1:** Characteristics of subjects and contrast-enhanced ultrasound (CEUS) parameters

Parameter	IgAN^a^	HSPN^a^	*P*-value^b^
No. of patients	13	12	
Male/female (No. of patients)	8/5	8/4	1.00
Age (years)	11.3 ± 2.6	10.4 ± 3.9	0.51
History (months)	3.8 ± 8.1	2.1 ± 2.1	0.47
Systolic blood pressure (mmHg)	116 ± 11	107 ± 5	**0.03**
Diastolic blood pressure (mmHg)	72 ± 10	68 ± 7	0.25
Laboratory data			
Albumin (g/L)	35.5 ± 5.4	35.7 ± 6.2	0.91
Total urinary protein (g/24 h)	2.0 ± 1.4	2.1 ± 1.7	0.83
uRBC/HP (No. of RBC)	40 ± 31	28 ± 20	0.30
Serum creatinine (μmol/L)	52.0 ± 13.9	49.3 ± 12.4	0.62
BUN (mmol/L)	6.9 ± 1.9	4.1 ± 1.3	**0.001**
Oxford classification (No. of patients)			
M0/M1	4/9	2/10	0.65
E0/E1	1/12	2/10	0.59
S0/S1	12/1	10/2	0.59
T0/T1	8/5	10/2	0.38
C0/C1/C2	2/7/4	3/7/2	0.66
Lee grading (No. of patients)			
II/III/IV	3/4/6	5/5/2	0.26
CEUS parameters			
IMAX (%)	100 ± 0	100 ± 0	1.00
RT (s)	25.3 ± 4.7	22.7 ± 1.9	0.09
TTP (s)	26.4 ± 5.6	23.4 ± 2.3	0.09
mTT (s)	303.5 ± 129.0	360.3 ± 191.2	0.40
QOF (%)	86.1 ± 5.5	87.6 ± 3.5	0.43

*BUN* blood serum urea nitrogen, *HSPN* Henoch-Schönlein purpura nephritis, *IgAN* immunoglobulin A nephropathy, *IMAX* maximum intensity, *mTT* mean transit time, *No.* number, *QOF* quality of fit, *RT* rise time, *TTP* time to peak, *uRBC/HP* number of red blood cells per high power microscopic field

^a^ Values are expressed as number or mean ± standard deviation

^b^ *P* < 0.05 is significant (bold)

### Contrast-enhanced ultrasound parameters analysis

**Fig. 1:**
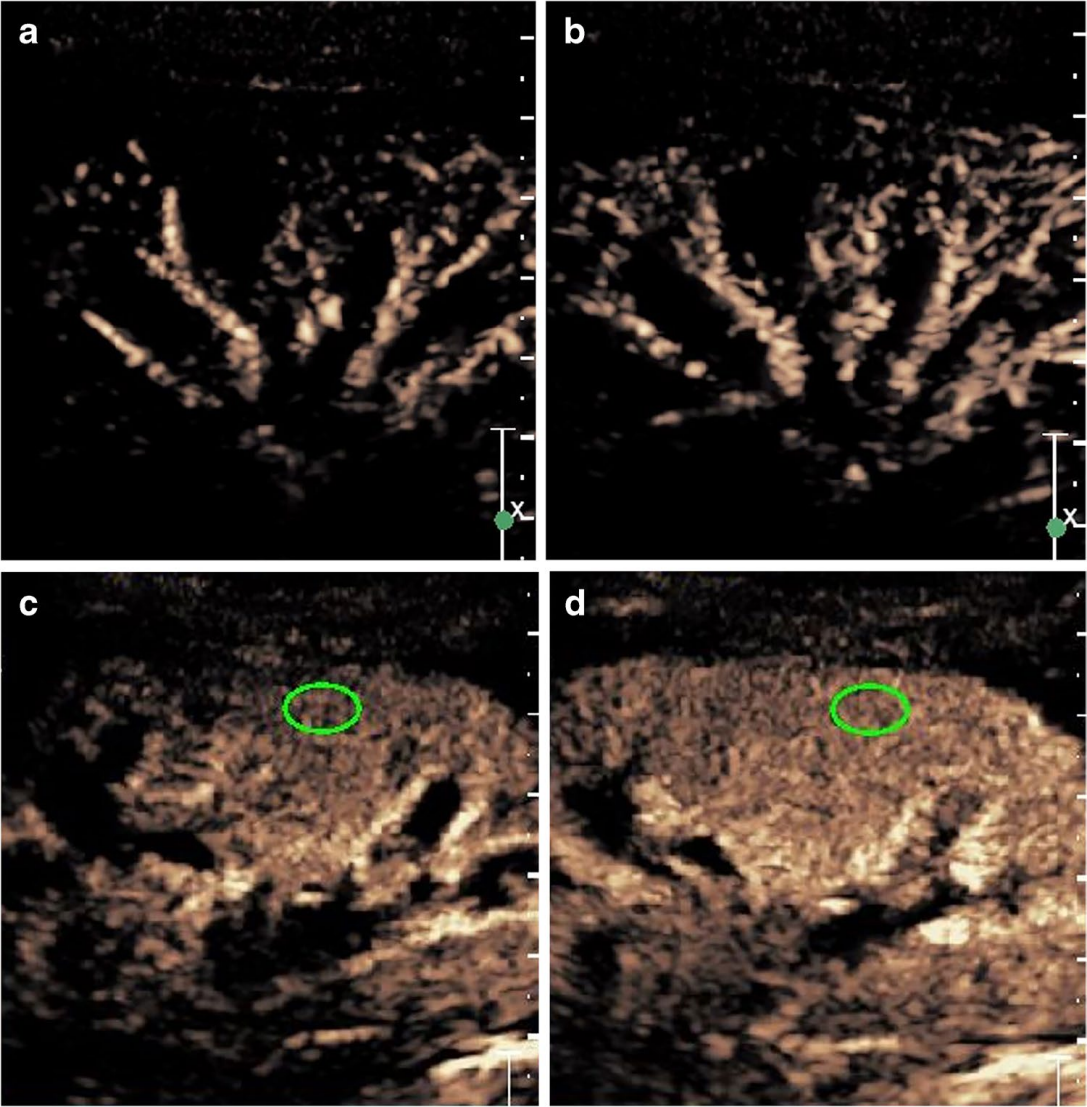
Representative longitudinal early phase real-time images of the kidney following a bolus injection of SonoVue in a 12-year-old girl with immunoglobulin A nephropathy. **a** Imaging at 14 s shows influx of contrast agent into interlobar arteries. **b** At 15 s, image shows contrast influx into arcuate arteries and start point of cortical enhancement. **c** At 21 s, there is medullary enhancement; *green oval* shows the region of interest (ROI) placement. **d** Imaging at 23 s shows maximal intensity of cortical enhancement; *green oval* shows the ROI placement

**Fig. 2:**
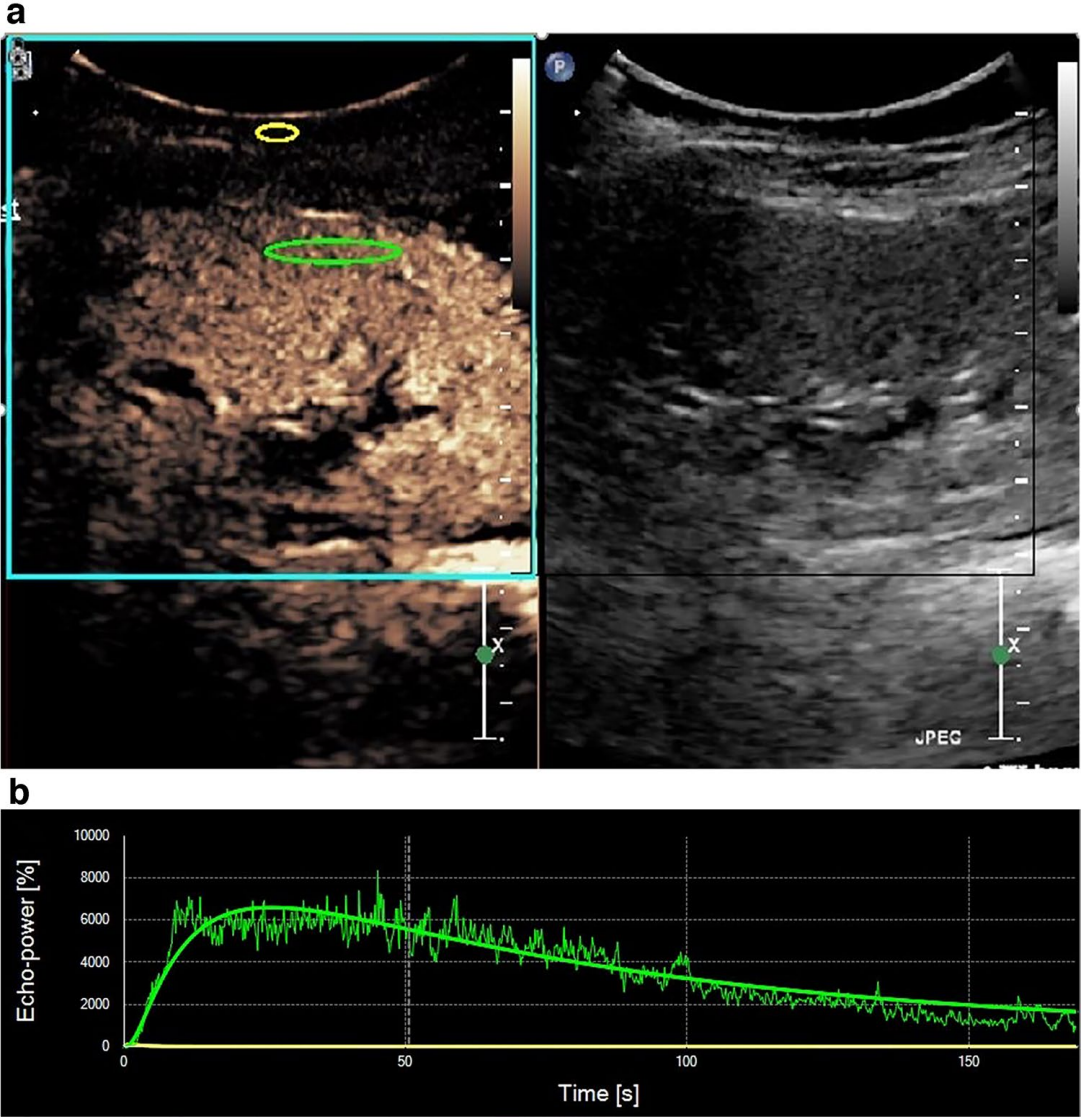
Time–intensity curve of renal cortex (immunoglobulin A nephropathy in a 7-year-old boy). The curve was drawn using the SonoLiver quantitative analysis software. **a** The *green oval* represents the region of interest in the renal cortical area. The *yellow oval* represents the reference region in the subcutaneous connective tissue. **b** The non-smooth green curve is the original curve, and the smooth green curve is the fitting curve. The *yellow line* is the curve of the reference region

**Fig. 3:**
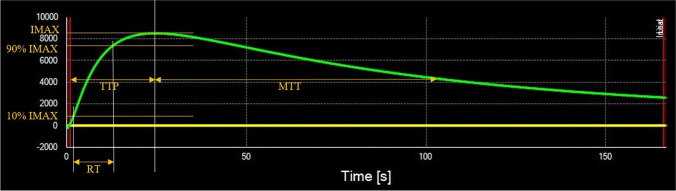
The quantitative parameters obtained from the time–intensity curve (*green line*). The *yellow line* represents the reference region. *mTT* mean transit time corresponding to the center of gravity, *RT* time between 10% and 90% of maximum intensity, *TTP* time to peak

After contrast medium was injected for CEUS, sequential enhancement of the renal interlobar arteries, arch arteries, renal cortex and renal medulla was observed in real time, followed by rapid peak intensity and slow decrease until resolution (Fig. 1). The time–intensity curves were generated automatically. Figure 2 shows a typical time–intensity curve obtained from a subject with IgAN. Figure 3 shows the quantitative parameters obtained from the time–intensity curve. The perfusion parameters showed no statistically significant differences in maximum intensity, rise time, time to peak, mean transit time and quality of fit values between the IgAN and HSPN groups (all *P* > 0.05). No child had adverse effects during the examination. The CEUS parameters in both groups are shown in Table 1. Renal enhancement following the bolus injection of SonoVue in an 11-year-old boy with IgAN is shown in Online Supplementary Material 7.

### Relationship between contrast-enhanced ultrasound data and Lee pathology grade

**Fig. 4:**
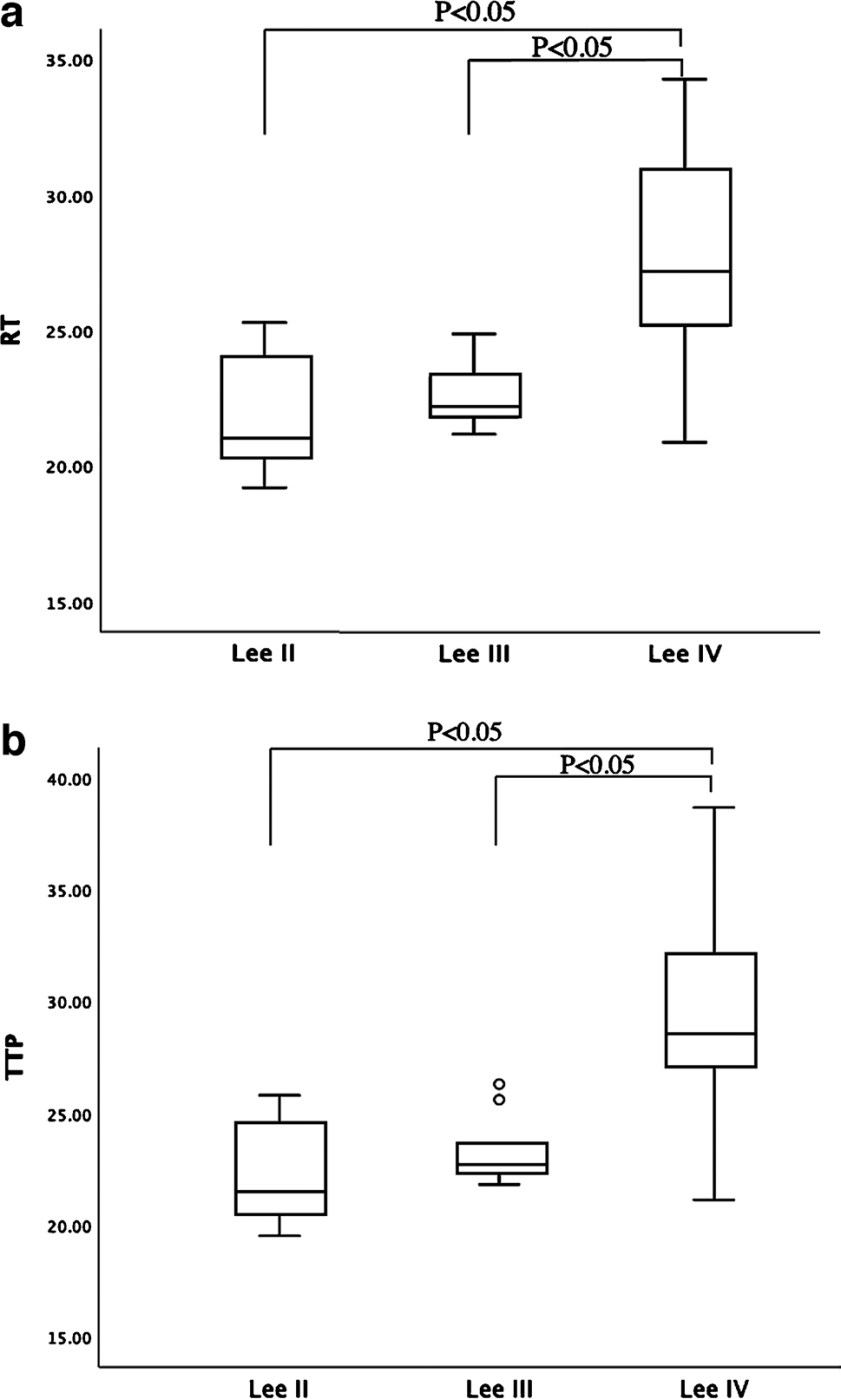
Box-and-whisker plot shows contrast-enhanced ultrasound (CEUS) parameters RT and TTP for different Lee grades. *RT* time between 10% and 90% of maximum intensity, *TTP* time to peak

In accordance with the Lee grading standard, all children with either IgAN or HSPN were divided into three groups: Lee II (*n* = 8), Lee III (*n* = 9) and Lee IV (*n* = 8). The CEUS parameters RT and TTP values increased gradually with increasing pathological grade and were significantly higher in the Lee IV group compared to those in the Lee II and Lee III groups (*P* < 0.05). However, we observed no statistical difference between children with Lee II and Lee III grades. The parameter of mTT decreased gradually with Lee pathological grade, although this trend was not statistically significant. The results are shown in Table 2 and Fig. 4.

**Table 2:** Relationship between contrast-enhanced ultrasound parameters and Lee grade

	Lee II	Lee III	Lee IV	*P*-value
No. of patients	8	9	8	
RT (s)	21.9 ± 2.3^a^	22.7 ± 1.3	27.7 ± 4.3^b^	**0.001**
TTP (s)	22.4 ± 2.4^a^	23.3 ± 1.6	29.4 ± 5.1^b^	**0.001**
mTT (s)	410.9 ± 172.6	322.0 ± 122.5	260.5 ± 169.7	0.11
QOF (%)	87.2 ± 4.7	88.1 ± 4.3	85.1 ± 4.9	0.25

*mTT* mean transit time, *QOF* quality of fit, *RT* rise time, *s* seconds, *TTP* time to peak

^a^ *P* < 0.05 (significant, bold), Lee II compared to the Lee IV

^b^ *P* < 0.05 (significant, bold), Lee III compared to the Lee IV

### Spearman correlation analysis

The Lee grading level correlated positively with the CEUS parameters RT and TTP (r = 0.552, *P* = 0.004; r = 0.583, *P* = 0.002, respectively) and negatively with mTT (r = –0.538, *P* = 0.006). Grade C in the MEST-C score correlated positively with the CEUS parameters RT and TTP (r = 0.487, *P* = 0.014; r = 0.504, *P* = 0.01, respectively). However, there was no significant correlation between the CEUS parameters and clinical data (age, blood pressure) and laboratory indices (urine protein excretion, level of microscopic hematuria, and serum albumin, creatinine, and urea nitrogen levels) (Table 3).

**Table 3:** Spearman correlation analysis

	RT		TTP		mTT	
	r	*P*-value^a^	r	*P*-value^a^	r	*P*-value^a^
Oxford classification						
M	–0.279	0.18	-0.279	0.18	0.019	0.92
E	0.282	0.17	0.231	0.27	0.043	0.84
S	-0.068	0.75	-0.145	0.49	0.256	0.22
T	0.124	0.56	0.136	0.52	–0.282	0.06
C	0.468	**0.02**	0.476	**0.02**	–0.201	0.34
Lee grading	0.628	**0.001**	0.663	**0.00**	–0.489	**0.01**
Age	0.036	0.86	–0.030	0.89	–0.056	0.78
Systolic blood pressure	0.116	0.58	0.076	0.72	0.245	0.24
Diastolic blood pressure	0.202	0.33	0.178	0.39	0.321	0.12
Albumin	0.125	0.55	0.147	0.48	–0.124	0.55
Total urinary protein	–0.236	0.26	–0.226	0.28	0.175	0.40
uRBC/HP	0.255	0.22	0.248	0.23	–0.248	0.23
Serum creatinine	0.137	0.51	0.078	0.71	–0.175	0.40
BUN	0.257	0.22	0.231	0.27	–0.111	0.60

*BUN* blood serum urea nitrogen, *mTT* mean transit time, *RT* rise time, *TTP* time to peak, *uRBC/HP* number of urine red blood cells per high power microscopic field

^a^ *P* < 0.05 is significant (bold)

### Relationship between the contrast-enhanced ultrasound data and MEST-C score

All children were divided into three groups according to their cellular/fibrocellular crescents formation score: C0 (*n* = 5), C1 (*n* = 14), C2 (*n* = 6). The CEUS parameters RT and TTP values gradually increased with the C grade and were significantly higher in the C2 group than in C0 and C1 groups (*P* < 0.05). However, we observed no statistical difference between C0 and C1. The results are shown in Table 4. There were no significant differences in CEUS parameters among different M, E, S or T score groups (Table 5).

**Table 4:** Contrast-enhanced ultrasound parameters according to different C scores of the Oxford classification

	C0	C1	C2	*P*-value
No. of patients	5	14	6	
RT (s)	22.1 ± 2.6^a^	23.4 ± 3.2	27.2 ± 4.5^b^	0.05
TTP (s)	22.8 ± 3.0^a^	24.1 ± 3.5	28.8 ± 5.9^b^	0.04
mTT (s)	329.0 ± 175.8	340.1 ± 136.9	330.8 ± 161.0	0.94
QOF (%)	86.3 ± 4.0	87.7 ± 5.0	85.3 ± 4.5	0.58

*mTT* mean transit time corresponding to the center of gravity, *QOF* quality of fit, *RT* time between 10 and 90% of maximum intensity, *s* seconds, *TTP* time to peak

^a^ *P* < 0.05, C0 compared to C2

^b^ *P* < 0.05, C1 compared to C2

**Table 5:** Contrast-enhanced ultrasound (CEUS) parameters according to M, E, S and T scores of the Oxford classification^a^

	M		E		S		T	
	M0	M1	E0	E1	S0	S1	T0	T1
No. of patients	6	19	3	22	22	3	18	7
RT (s)	26.1 ± 4.6	23.4 ± 3.4	21.6 ± 2.6	24.4 ± 3.8	24.2 ± 4.0	22.7 ± 2.3	23.9 ± 4.1	24.3 ± 3.1
TTP (s)	26.8 ± 4.8	24.4 ± 4.3	22.5 ± 3.5	25.3 ± 4.6	25.2 ± 4.7	23.1 ± 2.4	24.8 ± 4.9	25.4 ± 3.6
mTT (s)	304.3 ± 80.8	339.1 ± 180.1	326.8 ± 244.6	331.3 ± 154.6	317.8 ± 161.0	425.9 ± 152.7	367.7 ± 169.6	235.8 ± 86.9
QOF (%)	87.7 ± 4.1	86.6 ± 4.9	87.3 ± 5.1	86.8 ± 4.7	86.8 ± 4.8	87.6 ± 4.2	86.6 ± 4.2	87.6 ± 6.0

*mTT* mean transit time corresponding to the center of gravity, *No.* number, *QOF* quality of fit, *RT* time between 10% and 90% of maximum intensity, *s* seconds, *TTP* time to peak

^a^ Data presented as mean ± standard deviation

## Discussion

Immunoglobulin A nephropathy and HSPN are common glomerular diseases in children, and about one-third of cases slowly and progressively develop renal impairment. Searching for a noninvasive imaging method to determine the degree of renal damage early is therefore extremely important for improving the prognosis of these children. Changes in renal microvascular perfusion play a significant role in the progression of renal disease. In this study, we examined the characteristics of hemodynamic changes on CEUS in children with glomerular disease, with the aim of evaluating the clinical potential of CEUS to assess the pathological severity of IgAN and HSPN.

Contrast-enhanced US technology has ﻿been used to quantitatively measure cortical perfusion in renal disease [11, 12]. Intravenously administered SonoVue contrast medium has been approved by the United States Food and Drug Administration (FDA) for the diagnosis of liver diseases in children, but its use is still off-label for kidney disease. The intensity of the CEUS signal correlates with contrast microbubble concentration, which in turn correlates with tissue perfusion blood flow [13]. The kidney has an abundant blood supply, accounting for 90% of the whole kidney blood flow. After an intravenous bolus injection of US contrast agent, a large number of microbubbles rapidly perfuse into the renal cortex, resulting in obvious enhancement of the cortical signal and a rapid peak in the time–intensity curve. As the contrast agent circulates and metabolizes, the signal intensity gradually decreases until regression. This series of steps represents the entire process of renal cortical perfusion [14]. The study by Zhang et al. [15] showed that CEUS was a feasible tool for evaluating the degree of renal allograft fibrosis, while other studies showed that CEUS could be used to assess changes in renal perfusion in people with chronic kidney disease and diabetic kidney disease [16–18].

Many conditions lead to increased hemodynamic resistance in the renal microvasculature, including mesangial hypercellularity, segmental glomerulosclerosis, endocapillary hypercellularity, crescent formation, tubular atrophy and interstitial fibrosis, all of which contribute to a reduction in microvascular perfusion of the renal cortex. The study by Yang et al. [5] demonstrated that CEUS quantitative parameters such as peak intensity values decreased gradually with the progression of ﻿tubular atrophy and interstitial fibrosis. In other words, cortical perfusion gradually decreases with the increasing severity of renal pathology [5].^.^

In recent years, some scholars have analyzed the value of the Oxford classification for evaluating the clinical and prognostic aspects of HSPN [8, 11, 12, 19]. The study by Li et al. [9] also suggested that the Oxford classification might be suitable for assessing the clinical manifestations and prognosis of people with HSPN. To make the pathological data comparable and uniform, in our study we rescored the HSPN group using both the Lee grading standard and the Oxford classification. After finding no statistical difference in CEUS parameters between IgAN and HSPN, we pooled all the cases to perform a further analysis and found that there was a close correlation between renal pathology and the CEUS parameters. Our data showed that as the renal pathological grade progressed in children with IgAN and HSPN, RT and TTP gradually increased while mTT gradually decreased. These findings suggest that in cases of severe renal pathology, renal blood perfusion velocity gradually slows and the number of microbubbles entering the renal cortex decreases per unit time, resulting in reduced intensity. Our study also demonstrated that RT, TTP and mTT were related to the pathological grade of crescent formation. Crescent formation is an important pathological change associated with the activity of IgAN and HSPN. Glomerular perfusion is affected by crescent formation, leading to decreased renal cortical blood perfusion and an increase in RT and TTP. In our study, we did not find the influences of different degrees of mesangial or endothelial cell proliferation on CEUS indicators, probably because there were no histological extensive and severe mesangial or endothelial cell proliferation cases in our study, and CEUS was not sensitive enough to reflect this kind of injury. Previous studies have found that CEUS reflects the degree of renal tubular atrophy or interstitial fibrosis in adults with IgAN, but severe renal tubular fibrosis or interstitial fibrosis in children is rare and we had no case of T2 in this study, so the relationship between CEUS and tubular injury could not be determined.

Compared to other imaging examinations, MRI and contrast-enhanced MRI are time-consuming and expensive, and they have many contraindications for children with metallic implants or renal insufficiency. Today, multiparametric renal MRI appears to be a promising technique for exploring the renal blood flow change, including arterial spin labeling (ASL), diffusion-weighted imaging (DWI) and blood oxygen level–dependent (BOLD) MRI, but there is still a lack of research on nephritis in children [20]. Compared with MRI, CEUS has obvious advantages, including a low risk of allergic reaction, no radioactivity and easy application. The intravascular US contrast agent is metabolized through the pulmonary circulation, tolerable and without hepatorenal toxicity. Moreover, CEUS can be used to observe the whole process of enhancement dynamically in real time.

This study is the first to use CEUS to evaluate hemodynamic changes in pediatric glomerular diseases, but it has some limitations. First, children must cooperate effectively during CEUS because the probe must be kept steady manually to avoid respiratory movements influencing the measurements. Second, to ensure the stability of the measurements, all US examinations and imaging analyses were performed by one radiologist and therefore neither the﻿ generalizability of this technique nor interobserver variability of the quantitative data could be assessed. Third, our sample size was relatively small and a larger population study is required to validate our findings. In addition, it is possible to evaluate renal blood flow changes in combination with multiple US methods, such as power Doppler US, sound touch elastography or multiparametric MRI. Of course, based on the current findings, CEUS cannot replace pathological examination, which is still considered the gold standard for diagnosis, but it can be used as an important adjunct, especially for children with contraindications to renal puncture who require urgent evaluation of renal injury.

## Conclusion

The CEUS technique can noninvasively, easily and sensitively measure renal hemodynamic changes and assess the severity of pathology in children with IgAN or HSPN. We consider that CEUS technology has considerable future potential for quantitative analysis of glomerular lesions.

## Supplementary Information

Below is the link to the electronic supplementary material.

Online Supplementary Material 1: Scatter diagram of RT and Lee's Grade relationship (DOCX 1.65 MB)

Online Supplementary Material 2: Scatter diagram of TTP and Lee's Grade relationship (DOCX 1.65 MB)

Online Supplementary Material 3: Scatter diagram of mTT and Lee's Grade relationship (DOCX 1.65 MB)

Online Supplementary Material 4: Scatter diagram of RT and Grade C of the Oxford Classification (DOCX 1.65 MB)

Online Supplementary Material 5: Scatter diagram of TTP and Grade C of the Oxford Classification (DOCX 1.65 MB)

Online Supplementary Material 6: Sample size calculated by PASS software (DOCX 80.6 KB)

Online Supplementary Material 7: Longitudinal view renal CEUS after a bolus injection of SonoVue in an 11-year-old boy with immunoglobulin A nephropathy. The video shows the enhancement and resolution cycle (MP4 27.2 MB)
