# Supplementary figures and images for: Contrast-enhanced ultrasonography for assessing histopathology in pediatric immunoglobulin A nephropathy and Henoch–Schönlein purpura nephritis
Source: Pediatr Radiol. 2022 Jun 13;52(13):2575–83. doi: 10.1007/s00247-022-05399-3 (PMC9701653; doi:10.1007/s00247-022-05399-3)

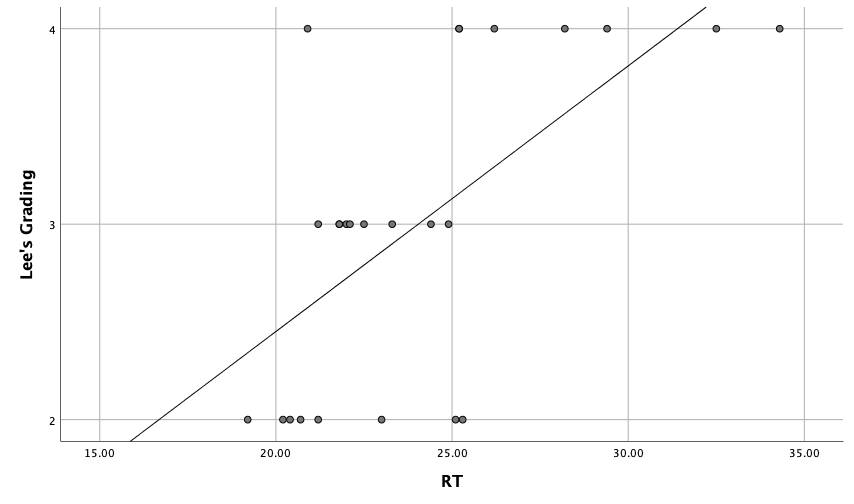


**Online Supplementary Material 1** Scatter diagram of (RT) and Lee’s Grade relationship

Supplement: Supplementary file 1 — Online Supplementary Material 1: Scatter diagram of RT and Lee's Grade relationship (DOCX 1.65 MB) [file 247_2022_5399_MOESM1_ESM.docx]

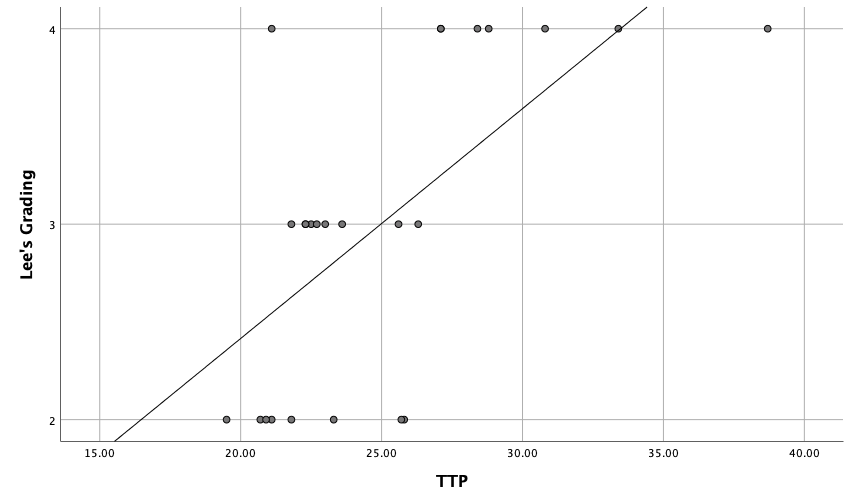


**Online Supplementary Material 2** Scatter diagram of TTP and Lee's Grade relationship

Supplement: Supplementary file 2 — Online Supplementary Material 2: Scatter diagram of TTP and Lee's Grade relationship (DOCX 1.65 MB) [file 247_2022_5399_MOESM2_ESM.docx]

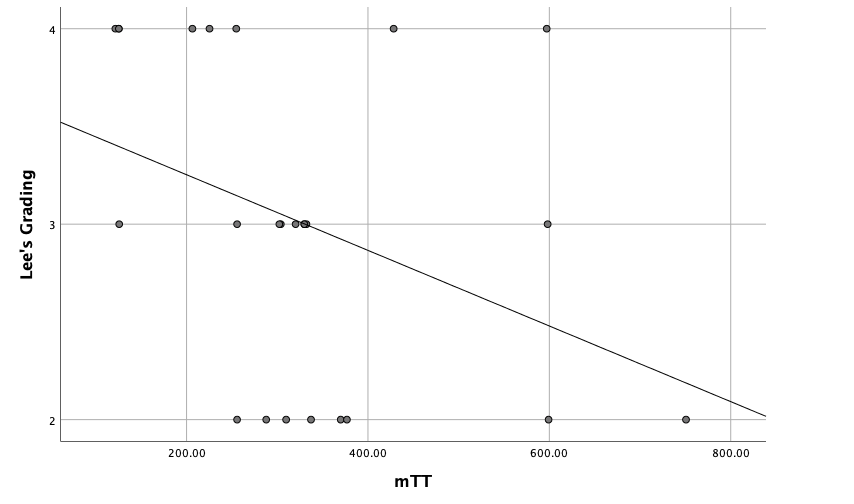


**Online Supplementary Material 3** Scatter diagram of mTT and Lee's Grade relationship

Supplement: Supplementary file 3 — Online Supplementary Material 3: Scatter diagram of mTT and Lee's Grade relationship (DOCX 1.65 MB) [file 247_2022_5399_MOESM3_ESM.docx]

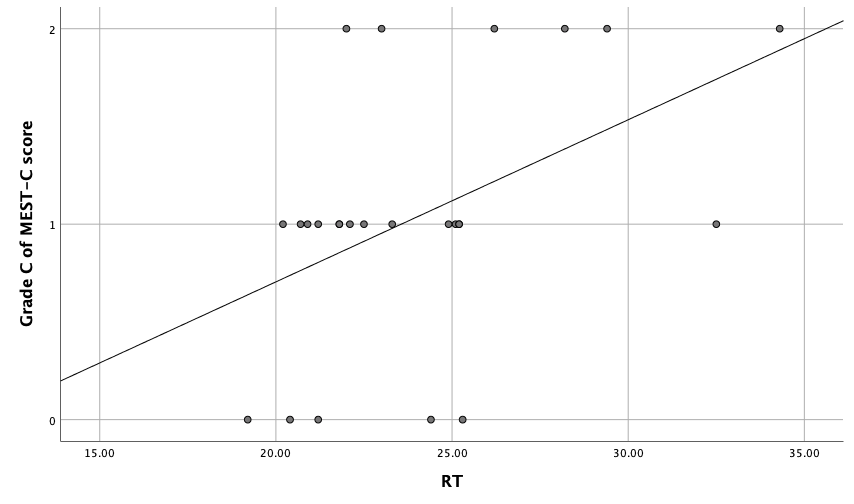


**Online Supplementary Material 4** Scatter diagram of RT and Grade C of the Oxford Classification

Supplement: Supplementary file 4 — Online Supplementary Material 4: Scatter diagram of RT and Grade C of the Oxford Classification (DOCX 1.65 MB) [file 247_2022_5399_MOESM4_ESM.docx]

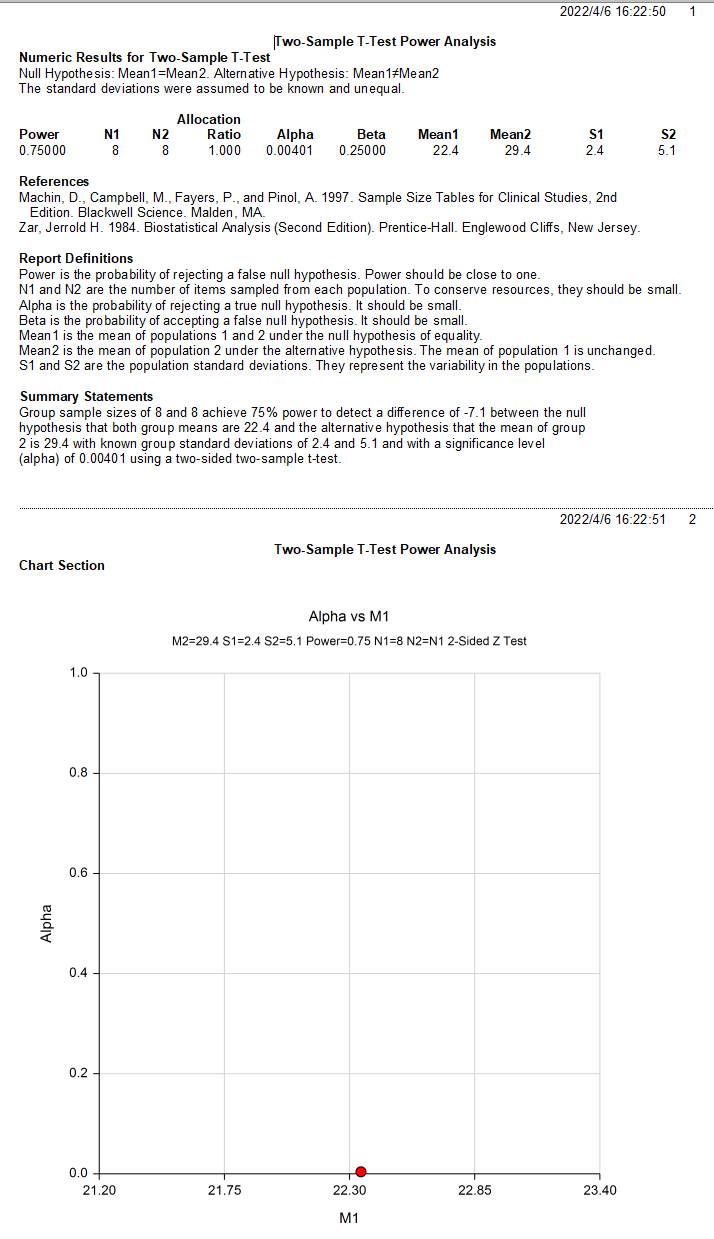


**Online Supplementary Material 6** Sample size calculated by PASS software

Supplement: Supplementary file 6 — Online Supplementary Material 6: Sample size calculated by PASS software (DOCX 80.6 KB) [file 247_2022_5399_MOESM6_ESM.docx]
