# Supplementary material for: Contrast-enhanced ultrasonography for assessing histopathology in pediatric immunoglobulin A nephropathy and Henoch–Schönlein purpura nephritis
Source: Pediatr Radiol. 2022 Jun 13;52(13):2575–83. doi: 10.1007/s00247-022-05399-3 (PMC9701653; doi:10.1007/s00247-022-05399-3)
Supplement: Supplementary file 5 — Online Supplementary Material 5: Scatter diagram of TTP and Grade C of the Oxford Classification (DOCX 1.65 MB) [file 247_2022_5399_MOESM5_ESM.docx]

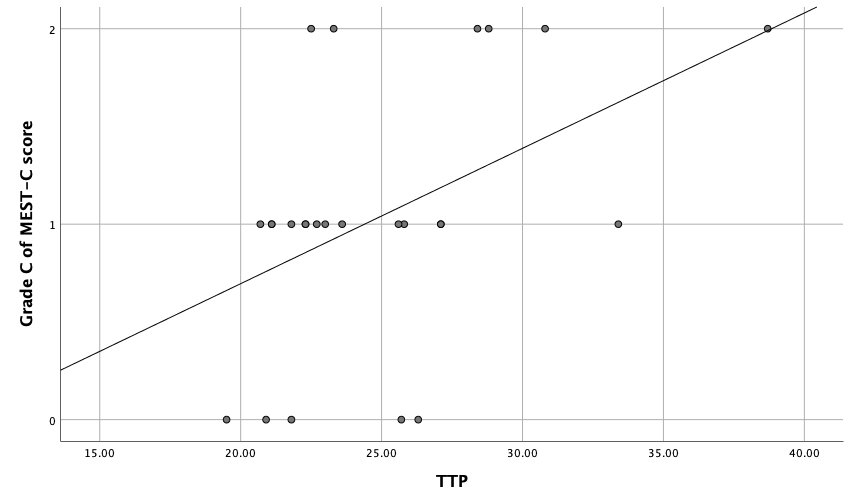


**Online Supplementary Material 5** Scatter diagram of TTP and Grade C of the Oxford Classification
